# Antropologia descolonizada: o papel de Portugal na produção antropológica entre o final do século XIX e meados do XX

**DOI:** 10.1590/S0104-59702024000100031

**Published:** 2024-07-15

**Authors:** Daniel Florence Giesbrecht

**Affiliations:** i Investigador, Centro de Estudos Interdisciplinares/Universidade de Coimbra.Coimbra – Portugal. profdanielflorence@gmail.com

No início do século XIX, o pensamento antropológico foi profundamente influenciado pelo desenvolvimento da antropologia física, que conquistou um posicionamento proeminente na Europa durante a segunda metade daquele século. Esse contexto conduziu a priorização de campos específicos de estudo ligados a genética, hereditariedade, somatologia e antropometria. Todos esses enfoques se direcionaram à exploração do tópico mais controverso dos primórdios da disciplina, que consistia na investigação das raças. Esse conceito de caráter puramente biológico, envolvendo a categorização e a classificação dos indivíduos em grupos raciais definidos por características físicas, transmissíveis e hereditárias, deixou marca duradoura na esfera da antropologia à medida que o novo século XX se desenrolava.

Pesquisadores de países como Inglaterra, França, Alemanha e Holanda lideraram esses estudos, e isso não foi por acaso, uma vez que essas nações adotaram políticas imperialistas no século XIX, visando conquistar e expandir os seus territórios coloniais nos continentes africano e asiático. No entanto, qual era a posição de Portugal, país pioneiro na expansão marítima e colonial, em relação às análises antropológicas dessas novas potências? Apesar de manterem importantes territórios coloniais na África e na Ásia, estariam os cientistas portugueses atrasados em suas pesquisas em comparação aos grandes centros europeus, a ponto de relegar Portugal a uma periferia no momento da institucionalização da antropologia?

Essas são apenas algumas das indagações, entre outras igualmente importantes, abordadas pela antropóloga portuguesa Patrícia Ferraz de Matos (2023), pesquisadora no Instituto de Ciências Sociais da Universidade de Lisboa, em seu livro recém-lançado *Anthropology, nationalism and colonialism: Mendes Correia and the Porto school of anthropology*, publicado pela conceituada editora Berghahn Books.

Desde o início, Patrícia apresenta *ad cautelam* suas principais abordagens metodológicas. Isso inclui a utilização de recursos provenientes da história e da antropologia para efetivamente alcançar seu objetivo central: examinar a vida dos antropólogos ou daqueles que foram reconhecidos como tal em sua época. Nesse contexto, a pesquisa se concentra especificamente em António Mendes Correia (1888-1960), médico, antropólogo, professor e principal divulgador da Escola de Antropologia do Porto. Essa decisão nos proporciona uma compreensão nítida da influência que os trabalhos de George Stocking (1966, 1982) exerceram sobre a autora, especialmente devido à proposição de que a avaliação das contribuições de antropólogos proeminentes do passado desempenha papel fundamental na revelação de narrativas e mitos associados à progressão da disciplina antropológica.

Por meio da figura de Mendes Correia, a obra sob análise alcança a maioria dos objetivos delineados. Um dos notáveis méritos reside na habilidade de estabelecer diálogo entre as fontes empregadas, cuidadosamente considerando as divergências como meio de fortalecer a base argumentativa. Métodos de análise hermenêutica, tais como dispositivos da linguística, semântica e semiótica, tornam-se discerníveis. O trabalho realizado por Patrícia Matos não somente resgata importantes fragmentos até então não revelados da antropologia portuguesa, íntimos ao contexto colonial do país – foi por meio da iniciativa de Mendes Correia que as missões antropológicas foram estabelecidas nas colônias Angola, Moçambique, Guiné-Bissau e Timor-Leste –, mas também se destaca pelo mérito significativo de descentralizar a narrativa da perspectiva histórica da antropologia europeia, explorando seu desenvolvimento em contextos nacionais. Ou seja, a biografia intelectual tornou-se apenas um caminho para a realização de algo muito maior e original, que abordou competentemente a transnacionalidade de seus objetos, que permitiram reconstituir, por meio das interações estabelecidas entre eles, de que forma o pensamento antropológico esteve presente em Portugal durante a era de Mendes Correia e como esse pensamento se relacionava não apenas com outros centros europeus, mas também com a América Latina (incluindo o Brasil) e os EUA.

O texto apresenta clareza e elegância, mantendo-se em sintonia com as expectativas de um trabalho acadêmico, evitando qualquer excesso de linguagem rebuscada. Isso o torna adequado à exigência científica e, ao mesmo tempo, garante que seja compreendido por uma audiência mais ampla. A estruturação é composta por cinco capítulos, incluindo a introdução e as considerações finais, as quais se assemelham mais ao formato de um capítulo adicional. Nelas, a autora compartilha suas impressões sobre a obra e introduz novos elementos para estimular aqueles que querem ampliar sua compreensão sobre questões cruciais, como a interconexão entre a evolução da antropologia portuguesa e os contextos de poder político e social.

No que diz respeito ao escopo temporal escolhido, que abrange desde o final do século XIX até a década de 1960, esse período foi caracterizado por debates intensos sobre as concepções raciais e nacionalistas. É essencial ressaltar que, independentemente do domínio disciplinar em que essas discussões emergiram (seja na medicina, na pedagogia ou no direito, por exemplo), a antropologia assumiu o papel de uma ciência fundamental, permeando diferentes epistemologias e servindo como alicerce para elucidar a maioria dos paradigmas raciais.

Na sua historicidade, a produção de Mendes Correia esteve intrinsecamente ligada às preocupações em torno das questões raciais e de higiene. Sob a égide de seu reconhecimento acadêmico, ele conseguiu abarcar diversas esferas, emitindo pareceres que se fundavam nas ciências médicas e biológicas a respeito de causas sociais e demográficas. Conforme apontado pela autora, ao analisarmos a vida e o legado de Mendes Correia, somos conduzidos ao entendimento das ideias discutidas na época, tanto em âmbito nacional quanto internacional, abarcando temas como problemas sanitários, saúde, alimentação e investigação científica. Além disso, essa análise lança luz sobre a complexidade de um período que atravessou duas guerras mundiais e culminou com a guerra colonial em Angola, em 1961.

Por último, mas não menos importante, a obra sublinha a relevância do “giro decolonial” para apreender os estereótipos e as formas como o Império Colonial Português refletia as ideias propagadas por seus antropólogos. As conjecturas, que vão desde a tentativa de separação entre os conceitos de raça e cultura, até a rejeição à miscigenação, foram elaboradas sob uma perspectiva eurocêntrica e racista. Quanto a isso, Edward Said (2007) já havia salientado a pertinência de compreendermos o colonialismo como uma tentativa de explicar, tipificar e hierarquizar povos e culturas, numa obsessão das potências coloniais para legitimar os seus domínios. Diante deste cenário, Patrícia Matos buscou evidenciar como Portugal, um país colonialista desde os primórdios da era moderna, lidava com tais questões.

Nota-se que, apesar de o Brasil não ser o foco principal do livro, sua relevância para o entendimento do percurso das ideias de Mendes Correia ao longo da vida é evidente. Esse fato, inclusive, já foi abordado em outro trabalho de Patrícia Matos (2013). Alinhado aos pressupostos racialistas da Escola Nina Rodrigues, de Sílvio Romero e de Oliveira Viana, Correia acreditava no branqueamento brasileiro. Para ele, a mestiçagem no processo de colonização portuguesa era um estratagema para manter a manutenção territorial. Só passou a considerar a miscigenação como um grande fator da expansão portuguesa depois da Segunda Guerra Mundial, o que, segundo ele, comprovaria a ausência de preconceito racial por parte dos colonizadores. A alteração de perspectiva de Mendes Correia é notoriamente influenciada pelos trabalhos culturalistas, com destaque para as contribuições do sociólogo Gilberto Freyre. Em sua segunda viagem ao Brasil, em 1937, Mendes Correia teve a oportunidade de se encontrar pessoalmente com Freyre, o que resultou em um estreitamento da relação entre eles.

*Anthropology, nationalism and colonialism: Mendes Correia and the Porto school of anthropology* é, portanto, uma leitura indispensável para aqueles que buscam compreender a conexão entre antropologia, nacionalismo e colonialismo na Europa do século XX. Patrícia Ferraz de Matos, mediante análise perspicaz e crítica, instiga o leitor a refletir sobre o passado, permitindo melhor compreensão do presente.
